# Quality of Antenatal Care in Primary Health Care Centers of Bangladesh

**Published:** 2014-12

**Authors:** Ahmed M. S. A. Mansur, Karim M. Rezaul, Hoque. M. Mahmudul, Chowdhury S

**Affiliations:** 1 Department of Community Medicine, Bangladesh University of Health Science, Dhaka, Bangladesh; 2 Department of Population Dynamics, National Institute of Preventive and Social Medicine (NIPSOM), Dhaka, Bangladesh; 3 Department of Community Medicine, National Institute of Preventive and Social Medicine (NIPSOM), Dhaka, Bangladesh

**Keywords:** Antenatal Care, Primigravida, Multigravida, Primary Health Care

## Abstract

**Objective:** To find out the quality of ANC in the Upazila Health Complexes (PHC centres) of Bangladesh.

**Materials and methods:** This cross sectional study was done in purposively selected three upazilas among the clients receiving antenatal care (ANC). Data were collected with questionnaire cum checklist in the context of two aspects of quality issues, namely assessment of physical arrangements for ANC (input) and services rendered by the providers (process).

**Results:** The mean age of respondents was 24.6±4.5 years. Majority of the respondents were with primary level education (60.3%). About half (52.8%) of the families had monthly income ranging from 3000-5000 taka (38-64 US$). Nearly half (48.9%) had no child, little more than one third (42.3%) were primigravida and 528 (57.7%) were multigravida. Out of 528 multigravid respondents 360 (68.2%) took ANC in their previous pregnancy whereas 168 (31.8%) did not take ANC Pregnancy outcome was found to be associated with receiving ANC (χ^2^=73.599; p=0.000). Respondents receiving ANC had more good pregnancy outcome. The mean waiting time for receiving ANC was 0.77±.49 hours. Out of the 13 centers, only 3 (23.1%) have sufficient instruments to render ANC services. Findings showed that where the modes of ANC service delivery in the ANC centers are fairly satisfactory. Though some of the points of standard operation procedures (SOPs) on ANC are not covered by some ANC centers, those were not considered necessary. But, regarding the physical facilities available for rendering ANC services, it is seen that facilities are not quite satisfactory. Number of doctors and nurses are not very satisfactory. One of the centers under this study has no doctor, where ANC services are given by nurses.

**Conclusion:** It can be concluded that the ANC services at the primary health care level is not adequate in Bangladesh. To ensure further improvement of the quality of ANC services, instruments used in logistics and supplies should be enhanced.

## Introduction

Systematic supervision (examination & advice) of a woman during pregnancy is called antenatal (prenatal) care (ANC). The care should start from the beginning of pregnancy and end at delivery. Antenatal care comprises of the following two factors: (i) History taking and examination (general and obstetrical) and (ii) Advice given to the pregnant woman.

The objective of antenatal care is to ensure a normal pregnancy with delivery of a healthy baby from a healthy mother (1). Insufficient maternal care during pregnancy and delivery are largely responsible each year for nearly 600,000 maternal deaths (2). The lowest rates of maternal care were found in Bangladesh, Chad, Mali, Nepal and Pakistan. (2). The level and practice of maternal mortality are important indicators of maternal health. Reliable statistics to monitor maternal health in Bangladesh is scarce and only limited information is available (3).

In a study on maternal and prenatal outcome in grand multiparty revealed that favorable outcome was observed in those patients receiving antenatal care in health delivery (4).

The researchers in a teaching hospital stated that factors which are responsible for antenatal death could be avoided by briefing the pregnant patients for antenatal checkup, detection of high risk pregnancy and early hospitalization of high risk patients (5).

It was revealed in some studies that the main factors behind high incidence of maternal morbidity and mortality were both poor access to antenatal care and delivery conduct by untrained personnel (6).

The maternal and child health-family planning (MCH-FP) project in Matlab - a rural area of Bangladesh- was conducted to evaluate the efficacy of obstetric care by midwives. During a three-year period after start of the maternity care program, the risk of obstetric death was significantly lower in the intervention area where by midwives provided health care services as compared to the control area where midwives provided no health care services (7).

The high number of maternal deaths in some areas of the world reflects inequities in access to health services, and highlights the gap between rich and poor. Almost all maternal deaths (99%) occur in developing countries (8). Unreliable information exists on levels of serious morbidity related to pregnancy and childbirth, or differentials between developed and developing countries (9). 

The major causes of maternal deaths were post partum hemorrhage (PPH) (37%), eclampsia (16%), and hepatic failure (11%) (10). MMR was reported to be 3.2 (BMMS, 2001), which showed a reduced rate of 2.4/1000 live births at mid of 2010 (11). This needs enhancement of ANC with use of standard operation procedures (SOPs). The aim of the research was to find out the quality of the ANC management in some of selected areas where the SOPs had been implemented and to compare with some of the selected areas where the SOPs had not been implemented.

## Materials and methods

This descriptive cross sectional research included 915 clients selected purposively from the ANC providing centers of three upazila health complexes (primary health care centers) and 10 home & family welfare centers (H&FWCs; Sub-centers) and union sub-centers. Data collecting instrument was a questionnaire cum checklist prepared on the basis of the SOPs developed by the Director General Health Services (DGHS). Pre-testing the questionnaire was done before the finalization of data collecting instrument. Data was collected using the direct interview and observation method by a data collector trained earlier on the process of data collection. At the end of each day of data collection, the editing of the filled-up instrument was done and necessary corrections were performed for any omission or incorrectness. Data was analyzed using Software Package Used for Statistical Analysis (SPSS) version 19.

## Results

Mean age of the respondents was 24.56 ±4.498 years. Maximum (399, 43.6%) respondents were within age range of 21-25 years. The age range of the respondents was 17 to 38 years Forty two percent (42.3%) respondents were primigravida, 31.5% respondents were in their 2^nd^ gravida and rest (26.2%) were multigravida. Also 35.4% of the respondents had the first ANC visit .264 (28.9%) respondents had second visit and rest (33.7%) had third or more number of visits. Out of 528 multigravid respondents, 360 (68.2%) chose ANC in their previous pregnancy, whereas 168 (31.8%) did not consider this center (Table 1). 

**Figure 1:**
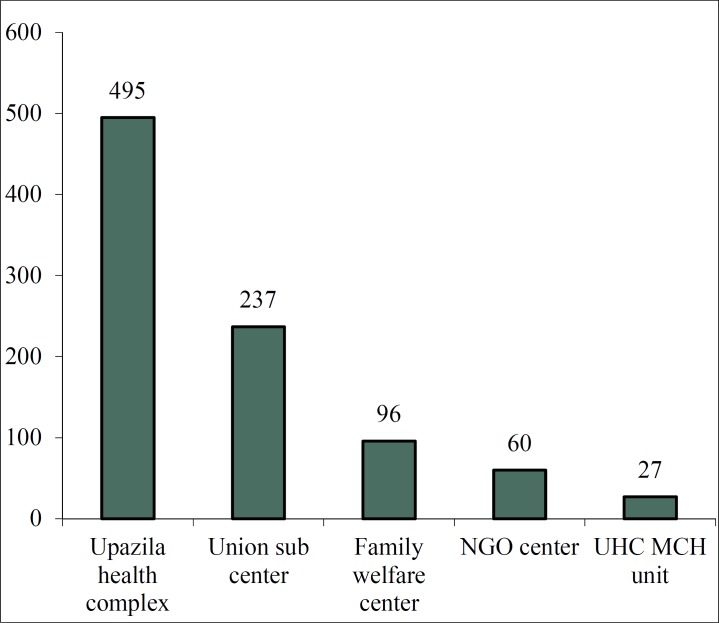
Distribution of the respondents by type of the centers

Figure 1 shows that there were 3 upazila health complexes (UHC) and 495 (54.1%) respondents were interviewed there. In addition, we interviewed 237 (25.9%) individuals from 6 union sub-centers, 96 (10.5%) individuals from 2 H&FWCS, 60 (6.6%) individuals from 1 NonGovermental organization managed Health center and 27 (3.0%) individuals from 1 upazila health complex unit for maternal and child health (MCH).

**Table 1:** Distribution of the respondents as per age, gravida and ANC related variables

Variables	Frequency	Percent
**Age group**		
** Up to 20 years**	**228**	**24.9**
** 21-25 years**	**399**	**43.6**
** 26-30 years**	**204**	**22.3**
** 31-35 years**	**75**	**8.2**
** > 35 years**	**9**	**1.0**
** Total**	**915**	**100.0**
**Gravida**		
** 1**	**387**	**42.3**
** 2**	**288**	**31.5**
** 3 or more **	**240**	**26.3**
** Total**	**915**	**100.0**
**Number of ANC visit**		
** 1**	**324**	**35.4**
** 2**	**264**	**28.9**
** 3 or more**	**327**	**35.7**
** Total**	**915**	**100.0**

Most of the ANC services and advices provided services more than 60% of the respondents. Low rate was observed in case of height measurement, checking ankle edema, enquiring of per vaginal bleeding, checking fetal heart sound and advising about signs of high risk pregnancy (Table 2).

As regards superstructures, out of 13 centers, sign board was present in 10 (76.9%) centers, but board showing time schedule of delivering ANC was absent in 8 (61.5%) centers. Toilet facility for the patients and their attendant was present in 11 (84.6%) centers. But, the condition of the toilet was not satisfactory in 7 (63.6%) of these 11 centers. Running water was not available in 6 (46.2%) centers. Privacy was maintained in all the centers (Table 3).

**Table 2:** Distribution of the respondents by receiving services and advices in relation to ANC

Variables	Frequency (%)		
Services and advices	Yes	No	Do not know / cannot remember
**Measured height? (n = 915)**	**390 (42.6%)**	**522 (57.0%)**	**0 (0.0%)**
**Measured weight? (n = 915)**	**714 (78.0%)**	**201 (22.0%)**	**0 (0.0%)**
**Measured BP? (n = 915)**	**879 (96.1%)**	**36 (3.9%)**	**0 (0.0%)**
**Saw anemia in eyelid? (n = 915)**	**618 (67.5%)**	**297 (32.5%)**	**0 (0.0%)**
**Saw edema in ankle? (n = 915)**	**546 (59.7%)**	**357 (39.0%)**	**12 (1.3%)**
**Supplied iron tablet? (n = 915)**	**867 (94.8%)**	**48 (5.2%)**	**0 (0.0%)**
**LMP enquired? (n = 915)**	**885 (96.7%)**	**27 (3.0%)**	**3 (0.3%)**
**EDD informed? (n = 915)**	**855 (93.4%)**	**54 (5.9%)**	**6 (0.7%)**
**PV bleeding enquired? (n = 915)**	**495 (54.1%)**	**408 (44.6%)**	**12 (1.3%)**
**Measured Uterine height? (n = 915)**	**801 (87.5%)**	**108 (11.8%)**	**6 (0.7%)**
**Heard Fetal Heart sound? (n = 915)**	**519 (56.7%)**	**378 (41.3%)**	**18 (2.0%)**
**Advice on high risk pregnancy? (n = 915)**	**501 (54.8%)**	**402 (43.9%)**	**12 (1.3%)**
**Advice on Family Planning? (n = 915)**	**554 (60.5%)**	**337 (36.8%)**	**24 (2.6%)**
**Advice on breast feeding? (n = 915)**	**813 (88.9%)**	**102 (11.1%)**	**0 (0.0%)**
**Advice on nutritious food? (n = 915)**	**828 (90.5%)**	**69 (7.5%)**	**18 (2.0%)**
**Advice on personal hygiene (n = 915)**	**651 (71.1%)**	**249 (27.2%)**	**15 (1.6%)**
**Advice on vaccinate child? (n = 915)**	**768 (83.9%)**	**144 (15.7%)**	**3 (0.3%)**
**Advice on next visit? (n = 915)**	**813 (88.9%)**	**102 (11.1%)**	**0 (0.0%)**

Out of the 13 centers, only 3 (23.1%) have sufficient instruments to render ANC services. Height measuring scale was not available in 9 (69.2%). Blood pressure (BP) machine was not available in 2 (15.4%) centers, and surprisingly, one center did not have stethoscope, where as two centers did not have thermometer**.** Ambulance was not available in 8 (61.5%) centers. With regards to lab investigation reports, five (38.5%) centers had urine albumin detection facility, and 5 (38.5%) centers had hemoglobin estimation facility. Iron and folic distribution to the pregnant women was found in all the centers (Table 4).

**Table 3:** Distribution of the ANC providing centers by superstructures and related facilities

Variables	Frequency	Percent
**Sign board**	**No.**	**%**
** Present**	**10**	**76.9**
** Absent**	**3**	**23.1**
**Time schedule board**		
** Present**	**5**	**38.5**
** Absent**	**8**	**61.5**
**No. of ANC room**		
** 1**	**10**	**76.9**
** More than 1**	**3**	**23.1**
**Waiting room**		
** Present**	**8**	**61.5**
** Absent**	**5**	**38.5**
**Toilets for patients & attendants**		
** Present**	**11**	**84.6**
**Absent**	**2**	**15.4**
**Toilet condition**		
** Satisfactory**	**4**	**36.4**
** Unsatisfactory**	**7**	**63.6**
**Running water**		
** Present**	**7**	**53.8**
** Absent**	**6**	**46.2**
**Facility for maintaining privacy**		
** Yes**	**13**	**100.0**

Nine (69.2%) centers had one doctor to deliver ANC services. One center had 5 doctors for ANC and there was an NGO center. However, there was no doctor for giving ANC in one center. Eight (61.5%) centers were running with nurse. Numbers of nurses in rest of the centers were between 6 and 11. 

System of supervision was present in 9 (69.2%) centers. More than 53.8% (7) centers failed to fill up registration book completely, and unfortunately, in 3 (23.1%) centers, behavior of the service providers was not satisfactory.

**Table 4:** Distribution of the centers by availability of instruments, investigations materials and accessories

Variables	Frequency	Percentage
**Instrument**		
** Sufficient**	**3**	**23.1**
** Insufficient**	**10**	**76.9**
**TT injection facility**		
** Present**	**12**	**92.3**
** Absent**	**1**	**7.7**
**Height measuring scale**		
** Available**	**4**	**30.8**
** Not available**	**9**	**69.2**
**Weight measuring scale**		
** Available**	**11**	**84.6**
** Not available**	**2**	**15.4**
**BP machine**		
** Available**	**11**	**84.6**
** Not available**	**2**	**15.4**
**Stethoscope**		
** Available**	**12**	**92.3**
** Not available**	**1**	**7.7**
**Thermometer**		
** Available**	**10**	**76.9**
** Not available**	**3**	**23.1**
**Ambulance**		
** Present**	**5**	**38.5**
** Absent**	**8**	**61.5**
**Urine albumin detection facility**		
** Available**	**5**	**38.5**
** Not available**	**8**	**61.5**
**Hemoglobin estimation facility**		
** Available**	**5**	**38.5**
** Not available**	**8**	**61.5**
**Record in registration book**		
** Complete**		**46.2**
** Incomplete**		**53.8**
**Iron & folic acid tablets availability**	**13**	**100**

The majority (693, 75.7%) of the respondents opined that they had to wait for less than 1 hour to receive ANC, 198 (21.6%) respondents had to wait for 1-2 hours, and rest (2.6%) of respondents had to wait between 10 minutes and 2 hours and 30 minutes. The mean waiting time was 0.77 ± 0.49 hours.

## Discussion

The objectives of this study were to ascertain the services rendered by providers at H&FWC and Upazilla Health complex levels and to evaluate the physical facility available for the service delivery. Pregnant women came to receive ANC at 13 centers under 3 upazilas. The centers were upazila health complexes, union sub-centers, home and family warfare centers, UHC MCH unit and one NGO center. Altogether, 915 respondents were interviewed (Figure 1).

The mean age of the respondents was 24.6±4.5 years. Most respondents (43.6%) were within age range of 21-25 years followed by 17-20 years (24.9%) and 26-30 years (22.3%).Only 8.2% aged 31-35 years and 1% were above 35 years (Table 1). This finding is consistent with Khanam, a study done at NIPSOM, Bangladesh in 2007-08) (12). In her study, she found the mean age of the ANC seekers was 24.9 years, while minimum and maximum ages were 18 and 39 years, respectively. It seems to be that ANC service seekers are in group of young age mothers. 

Our findings showed that 42.3% experienced their first pregnancy, 31.5% experienced second gravida, 44% experienced primigravida, and 26.2% was multigravida which seems to be declined. It is comparable to results obtained in Iraq where 44% were primigravida (13). The declining trend in latter pregnancy may indicate presence of some barriers for accessing the ANC services. The study also shows that majority of the respondents (35.4%) were in their first ANC visit which is comparable with study done in Iraq in which showed that only 38% of women were in the first trimester during data collection (13). A study on maternal mortality in Bangladesh found that nearly 70% did not visit any place for ANC and only 30% visited health center for antenatal care (14). This may indicate women are not motivated to continue their ANC check-up (Table 1).

The result of the study also described services and advices in relation to ANC received by the respondents. It was seen that most of the services and advices were received by more than 60% of the respondents. Rates of height measurement, checking ankle edema, enquiring of PV bleeding, checking fetal heart sound and advising about signs of high risk pregnancy were slightly lower as compared to the other factors (Table 2). In study done by WHO, it is seen that diet and nutrition was among the most commonly discussed topic (47%). Regarding child spacing and family planning, 45% of clients reported receiving information on this topic which seem to be less than the related value of this study (15). In other study, less than 50% of participants reported that they were provided with IEC, and nearly 47% of women were advised about diet (13). In this study, 88.9% reported breast feeding was covered in health education, which is higher than related value reported in one study about breast feeding (34%) (16). These might indicate better patient-provider interactions regarding advices during ANC. 

The result also shows that sign board is present in 76.9% of centers, but time schedule of delivering ANC on the board was absent in 61.5% of centers. Toilet facility for the patients and their attendant was present in 84.6% centers. But, the condition of the toilet was not satisfactory in 63.6% of the centers. Running water was not available in 6 (46.2%) centers. Regarding condition of cleanliness, 69.2% had satisfactory environment. Privacy was maintained in all the centers. But in a study done in Iraq, it was found that nearly half (48%) of clients reported adequate privacy (13). This study may indicate relatively better quality of service compared the related values in Iraq (Table 3).

In this study, among 13 centers, only 3 (23.1%) have sufficient instruments to render ANC services. Height measuring scale was not available in 9 (69.2%). Blood pressure machine was not available in 2 (15.4%) centers, one center lacked stethoscope, and two centers did not have thermometer. These findings indicate inadequacy of the input in our study sites. However, in a study done in northwest Ethopia, it was seen that all health facilities had functional weight scale, microscope, fetoscope and stethoscope, but sphygmomanometer was not available in one health facility (17) (Table 4).

About less than two fifth (38.5%) centers had urinary albumin detection facility and the rest (38.5%) had hemoglobin estimation facility, suggesting inadequacy of service. However, iron and folic acid supplements were distributed to the pregnant women from all the centers. A study in northwest Ethiopia showed that iron sulfate/folic acid supplements was present only in one facility (17) (Table 4).

It was found that except one, all other centers had at least one doctor for ANC service. However, more than one doctor (ranging from 2-5) was present in 3 centers. This is comparable to results obtained in Medina which stated that having only1 physician for ANC services in a large primary health care center (PHCC) is unlikely to meet the recommended time for ANC which is supposed to be given for each woman to discuss her personal needs and for the physician to respond appropriately, especially on the first visit when a full history has to be taken and an individualized birth plan started (18). The result of this study has also indicated that there is an overall health worker shortage. This high shortage of health worker hinders the capacity of health system to deliver the required services to its clients. 

The study also showed that system of supervision system was present in 69.2% of centers. It may indicate to have a weak supervision system which in turn frustrated efforts to improve quality of ANC services. Unfortunately, in 23.1% of centers, behavior of the service providers was not satisfactory, but study done in Nigeria showed that the healthcare providers’ attitudes were perceived to be good by 66.3% of respondents, while 25.7% of clients felt providers’ attitudes were fair, whereas 8.0% felt healthcare providers had poor attitudes (19). This needs to be understood further 

It was found that the majority 693(75.7%) of the respondents opined that they had to wait for < 1 hour to receive ANC. But, study in Gujrat showed that the mean waiting time was similar both in the community surveys and in exit interviews, which was about 30 minutes (20). The waiting time correlates well with the average waiting time in similar settings (21-25). It also emerges as one of the major areas of dissatisfaction with the health services as well as the cause of non-utilization. A reduction in the waiting time could improve patient satisfaction and enhance the utilization of health services provided by the primary health center (20).

## Conclusion

Considering and analyzing the findings of this study, it is seen that the quality of ANC service delivery in the ANC centers are moderately satisfactory. Though some of the points of SOP on ANC are not covered by some ANC centers, those were not considered important. But, regarding the physical facilities available for rendering ANC services, it is seen that facilities are not quite satisfactory. Number of Doctors and Nurses are not also very satisfactory. Having more physicians available for ANC in PHCCs could improve the standard of care. Further studies could take a more in-depth look at physicians’ and nurses’ characteristics and opinions to examine factors they face in their work place, their suggestions for improving ANC and their job satisfaction.

## Confilict of Interests

Authors declare that they have no conflict of interest.

## References

[1] Dutta DC (1998). Textbook of Obstetrics.

[2] Antenatal care could save millions ( 2000). Data briefs: Progress and disparity, The Progress of Nations 2000.

[3] Hlady WG, Fauveau VA, Khan SA, Chakraborty J, Yunus M (1992-1993). Utilization ofmedically-trained birth attendants in rural Bangladesh. Asia Pac J Public Health.

[4] Rahman MM, Barkat-e-Khuda, Kane TT, Mozumder KA, Reza MM (1997). Determinants of antenatal care seeking behaviour in rural Bangladesh. Dhaka: International Centre for Diarrhoeal Disease Research, Bangladesh,.

[5] Ahmed FU, Rahman ME, Perveen R (1997). Factors associated with the utilization of trained traditional birth attendants in rural Bangladesh. TROPICAL DOCTOR.

[6] HPSP (1997). Preliminary Report. MOHFW.

[7] Harun Or Rashid Md (1998). Standards for Thana Hospitals, Development of Health Care Quality Assurance project. DGHS, Govt. of Bangladesh.

[8] Maternal mortality (2014). Fact sheet No.348.

[9] Rooney C (1992). Antenatal care and maternal health: - World Health Organization.

[10] Khatun F, Rasheed S, Moran AC, Alam AM, Shomik MS, Sultana M (2012). Causes of neonatal and maternal deaths in Dhaka slums:implications for service delivery. BMC Public Health.

[11] HNPSP (2003-2010). Government of the People’s Republic of Bangladesh.

[12] Khanam US (2007). Attitude of the pregnant women towards antenatal care at Gazipur Sadar Hospital. Dissertation [NIPSOM].

[13] Awring MR, Al-hadithi TS (2011). Antenatal care in Erbil city-Iraq: Assessment Of Information, Education And Communication Strategy. Duhok Medical Journal.

[14] Akhter HH, Chowdhury ME, Sen A (1996). A Cross Sectional Study on Maternal Morbidity in Bangladesh. Institute Of Research for Essential and Reproductive Health and Technologies.

[15] WHO (2002). WHO antenatal care randomized trial: manual for the implementation of the new model. UNDP/UNFPA/WHO/World Bank Special Programme of Research, Development and Research Training in Human reproduction.

[16] Banerjee B (2009). Information, Education, and Communication Services in MCH Care Provided at an Urban Health Center. Indian J Community Med.

[17] Ejigu T, Woldie M, Kifle Y (2013). Quality of antenatal care services at public health facilities of Bahir-Dar special zone, Northwest Ethiopia. BMC Health Serv Res..

[18] Habib F, Hanafi MI, El-Sagheer A (2011). Antenatal care in primary health care centres in Medina, Saudi Arabia, 2009: a cross-sectional study. East Mediterr Health J.

[19] Sholeye OO, Abosede OA, Jeminusi OA (2013). Client Perception of Antenatal Care Services at Primary Health Centers in an Urban Area of Lagos, Nigeria. World Journal of Medical Sciences.

[20] Chandwani H, Jivarajani P, Jivarajani H (2008). Community Perception and Client Satisfaction about the Primary Health Care Services in a Tribal Setting of Gujarat – India. Internet journal of Health.

[21] Pradhan PMS, Bhattarai S, Paudel IS, Gaurav K, Pokharel PK (2013). Factors Contributing to Antenatal Care and Delivery Prac-tices in Village Development Committees of Ilam District, Nepal. Kathmandu University Medical Journal.

[22] Mendoza Aldana J, Piechulek H, al-Sabir A (2001). Client satisfaction and quality of health care in rural Bangladesh. Bull World Health Organ.

[23] Baltussen RM, Yé Y, Haddad S, Sauerborn RS (2002). Perceived quality of care ofprimary health care services in Burkina Faso. Health Policy Plan.

[24] Prasad B, Gupta VM (1999). A qualitative assessment of antenatal care provided by auxiliary nurse midwives. Indian J Public Health.

[25] El Shabrawy Ali M (1992). A study of patient satisfaction as an evaluation parameter for utilization of primary health care services. J R Soc Health.

